# Two-Dimensional Photoacoustic/Ultrasonic Endoscopic Imaging Based on a Line-Focused Transducer

**DOI:** 10.3389/fbioe.2021.807633

**Published:** 2022-01-06

**Authors:** Weiran Pang, Yongjun Wang, Lili Guo, Bo Wang, Puxiang Lai, Jiaying Xiao

**Affiliations:** ^1^ Department of Biomedical Engineering, School of Basic Medical Science, Central South University, Changsha, China; ^2^ Department of Biomedical Engineering, The Hong Kong Polytechnic University, Hong Kong, Hong Kong SAR, China; ^3^ Department of Biomedical Engineering, College of Biology, Hunan University, Changsha, China

**Keywords:** photoacoustic imaging (PAI), ultrasonic imaging (USI), acoustic resolution photoacoustic/ultrasonic endoscopy (PA/USE), line-focused transducer, improved back-projection imaging algorithm, imaging depth of field

## Abstract

Existing acoustic-resolution photoacoustic/ultrasonic endoscopy (PA/USE) generally employs a point-focused transducer for ultrasound detection, which is only sensitive in its focal region, thus the lateral resolution and sensitivity drop dramatically when the targets move far from its focus. Even if a dynamic focusing algorithm is applied, the sensitivity out of the transducer focus is still much lower than that in the focus in ultrasonic imaging mode. In this work, we propose an acoustic-resolution PA/USE with a line-focused transducer to realize automatic focusing for the first time. In comparison to a point-focused transducer, the line-focused transducer emits a more uniform sound field, causing the original signal intensity and signal-to-noise ratio (SNR) of the front and rear targets to be closer in the radial direction, which is beneficial for improving target signal uniformity in ultrasonic imaging. Simultaneously, we improved the resolution of the defocus area by modifying a prior work of back-projection (BP) reconstruction algorithm typically used in point-focused transducer based PAE and applying it to line-focused PA/USE. This combined approach may significantly enhance the depth of field of ultrasonic imaging and the resolution of the defocus zone in PA/US imaging, compared to the conventional method. Sufficient numerical simulations and phantom experiments were performed to verify this method. The results show that our method can effectively improve the lateral resolution in the image’s defocused region to achieve automatic focusing and perfectly solve the defect of the target signal difference in the far-focus region in ultrasonic imaging, while also enhancing the image SNR and contrast. The proposed method in this paper lays foundations for the realization of photoacoustic/ultrasonic combined endoscopy with enhanced lateral resolution and depth of field, which can potentially benefit a many of biomedical applications.

## 1 Introduction

Photoacoustic imaging (PAI) is an emerging non-invasive biomedical imaging technology. It uses a pulsed laser as the excitation source and provides the light absorption distribution inside biological tissue by detecting ultrasound generated from biological tissue due to the absorption of laser energy (Xu and Wang, 2006; Beard, 2011; Lai et al., 2015; Wang et al., 2016; Zhou et al., 2019). Because cancerous sites are usually accompanied by a large number of abnormal vascular hyperplasia, and hemoglobin in blood vessels is the main absorber of near-infrared light commonly used in PAI, the signals of cancerous sites in PAI are usually significantly stronger than those of normal tissues (Hoelen et al., 1998; Mallidi et al., 2011; Jnawali et al., 2020). Therefore, PAI has a very high detection sensitivity and specificity for cancerous tissue. At the same time, because photoacoustic imaging uses ultrasound, which is less scattered by the tissue, as the detection method, it has a high penetration depth and moderate spatial resolution, which are not available in ordinary optical imaging methods (Liu et al., 2016; Wang et al., 2016; Fu et al., 2019; Zhou et al., 2020; Chen et al., 2021).

Photoacoustic endoscopy (PAE) (Yang et al., 2012b; Yoon and Cho, 2013; Tang et al., 2016; Guo et al., 2020) is one of the forms of PAI, and acoustic resolution-based photoacoustic endoscopy (AR-PAE) combines the high tissue sensitivity of optical imaging, as well as high lateral resolution and high penetration depth of ultrasonic imaging. The lateral resolution can reach about 150 *μ*m, and it allows sub-centimeter deep detection (He et al., 2016; Wang et al., 2018). At present, AR-PAE has been used in endoscopic detection for animal esophagus and rectum (Yang et al., 2012b; Guo et al., 2020). In addition, PAE can also be easily integrated with ultrasonic imaging (USI) to form acoustic-resolution dual-mode photoacoustic/ultrasonic endoscopy (PA/USE) imaging (Yang et al., 2012b; James et al., 2014; Li et al., 2018). For example, in cervical cancer, a large amount of vascular hyperplasia are associated with the cancerous location, which will cause changes in tissue elastic dynamics and acoustic parameters in the region. The acoustic anatomic structure information of cervical tissue provided by ultrasonic endoscopic imaging will not only help to locate the functional information obtained by photoacoustic imaging, but may also provide additional acoustic tissue contrast for the diagnosis of cervical endocervical lesions. Therefore, acoustic-resolution dual-mode PA/USE has shown promising clinical applicability.

Currently, many explorations in the field have focused on reducing the size of the photoacoustic endoscope probe (Yang et al., 2012a; Ansari et al., 2015; Miranda et al., 2020), improving the resolution at the focal point (Cai et al., 2017; Wang et al., 2021), enhancing the sensitivity (Chen et al., 2015) and expanding the applications (Guo et al., 2020). For example, in order to obtain a tight focal resolution, point-focused high-frequency ultrasonic transducers are usually desired. However, only in the paraxial area near the focal zone, ultrasonic propagation can be approximated as a straight line, which means that a good lateral resolution can be obtained only near the focal region. When the target is far away from the focus, the lateral resolution decreases rapidly, seriously limiting the imaging depth of the field (Wang et al., 2021). To improve the depth of the field, it is necessary to use a longer focal length or a low-frequency ultrasonic transducer, which is bound to cause the reduction of lateral resolution at the focal point. Moreover, in ultrasonic imaging, the transmitting sound field of the point-focused transducer is very uneven, meaning that the ultrasonic trigger signal cannot effectively reach the target in the far-focus region, and the feedback ultrasonic signal is correspondingly small or almost non-existent, which again seriously limit the depth of the field as well as the image contrast. Therefore, to solve the irreconcilable contradiction between the lateral resolution and the imaging depth of field in PA/USE, it is urgent to develop an appropriate method to consider the influence due to the use of a point-focused transducer.

In this work, an acoustic-resolution PA/USE method based on a line-focused transducer is inspired, and the system is integrated with an improved back-projection imaging algorithm to achieve autofocusing and co-registration of photoacoustic/ultrasonic signals. The feasibility of this method was investigated through numerical simulations and phantom experiments, demonstrating significantly enhanced 2D imaging performance. In the next phase of study, the method will be further extended to achieve 3D ultra-high resolution PA/USE imaging.

## 2 Materials and Methods

### 2.1 Methods

**FIGURE 1:**
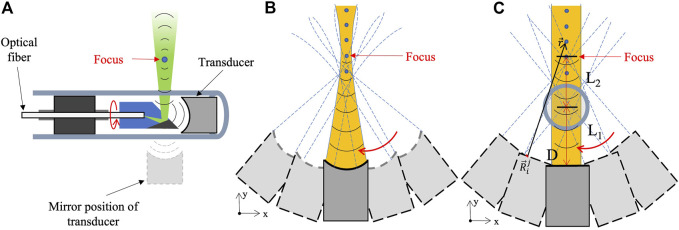
**(A)** Structure diagram of typical acoustic-resolution PA/USE probe; **(B)** Emission sound field and scanning path in the *X*–*Y* direction of a point-focused transducer; **(C)** Emission sound field and scanning path in the *X*–*Y* direction of a line-focused transducer.

The conventional acoustic resolution PA/USE technique usually uses a point-focused transducer and rotates the reflector module to achieve circular scanning. Figure 1A shows the structure of a typical PA/USE probe and its scanning method. The probe consists of a transparent hard shell, a focused transducer, a multi-mode fiber or single-mode fiber, a reflecting mirror, and an external driving device. The ultrasonic transducer here is not only used as a PA/US signal receiver (black solid line for echo), but also as an ultrasonic signal transmitter. Since the transducer is point focused, its sound field is first contracted and then diverged (yellow sound field and gradually shrinking isometric sound field lines), as illustrated in Figure 1B, which may cause ultrasound to not travel in a straight line and only be considered as a straight line near the focal area. So, in ultrasonic imaging, the target far behind the focus cannot receive effective ultrasonic excitation, which greatly reduces the detection sensitivity of the signal and leads to a serious reduction in the imaging depth of field. Moreover, when using a traditional BP algorithm to reconstruct the image, the target can only be clearly focused near the focal point, and when the target is far away from the focus, the lateral resolution will be seriously reduced both in photoacoustic and ultrasonic imaging. Therefore, it is critical to find a method to overcome these shortcomings in PA/USE imaging.

In this study, we suggest replacing the standard point-focused transducer with a line-focused one, in which a cylindrical lens is employed instead of a spherical lens, with a focal line in the X–Y plane and a detection surface that is nearly a plane in the *X*–*Y* direction. Then, we combine this with an improved back-projection imaging algorithm to perform high-resolution dynamic focusing. As shown in Figure 1C, the sound field of the line-focused transducer in the *X*–*Y* direction is more uniform than that of the point-focused transducer. This method has the following advantages. Firstly, compared to the point-focused transducer, the line-focused transducer has a more uniform ultrasonic emission field in the X–Y plane (the yellow sound field and straight-line propagating sound waves), so it can greatly enhance the detection sensitivity of the target outside the focus in ultrasonic endoscopic imaging, and thus greatly increase the depth of field. Secondly, since the line-focused transducer is a linear model in the X–Y plane, the back-projection algorithm of the regular linear model transducer can be used for calculation, which greatly improves the calculation efficiency and imaging speed of the algorithm. The scanning mode of the line-focused transducer in two dimensions is also shown in Figure 1C. Here, we set the distance between the center of the transducer and the center of the mirror as L1, the distance between the center of the mirror and the focus of the transducer as L2, then the focal length of the transducer is (L1+L2), and the diameter of the transducer is D. For circumferential side-view scanning, this is equivalent to placing the ultrasonic transducer in its mirror position and scanning along a circular path of radius L1.

### 2.2 Algorithm

Traditional back-projection (BP) algorithms are commonly employed in photoacoustic tomography because to their simplicity, robustness, and adaptability (Yang et al., 2005; Gamelin et al., 2009; Huang et al., 2015), and they have recently been used in PAE (Cai et al., 2017) we modified a previous work on improved BP algorithm (Wang et al., 2018) and applied it to line-focused transducer-based PA/USE. Rather than merely back aligning the A-line photoacoustic signals, the suggested approach analyzes the contribution of transducers at many points to provide the reconstructed value of one pixel in this study. This approach also takes into account the shape of the transducer’s detecting surface. In our algorithm, the ultrasonic transducer detection surface is divided into n tiny units by comprehensively considering the shape and size of the ultrasonic transducer detection surface and influence of the transducer position scanning at different angles on the resolution of any point in the image, which each unit treats as a point detector (*j* ^th^ detector, numbers from 1 to n). m is the number of transducer positions in a full circular scan (*i* ^th^ position detector, numbers from 1 to m). Since the detection surface is planar in the *X*–*Y* direction, the following formula can be used for imaging in combination with the principle of photoacoustic imaging:

$$I\left(r,\theta \right)=\overset{m}{\underset{\varphi =1}{\sum }}\left(S_{\varphi }\left(R_{\varphi }\left(r,\theta \right)/\vartheta \right)\right)$$ (1)

$$I\left({\vec{r}}^{\prime }\right)=A\times S_{i}\left(|{\vec{r}}^{\prime }-{\vec{R}}_{i}^{j}|/\vartheta \right)$$ (2)

where *S*_*φ*_(t) is the time domain sound pressure signal received when the ultrasonic detector rotates to the angle of *φ*, and $\left(R_{\varphi }\right.\left(r,\theta \right)$ is the distance between the point on the image plane coordinate (*r*, *θ*) and the detection surface of the plane ultrasonic transducer rotated to the angle of *φ*. We regard the position of point detector j (j = 1⋯ n) in the ith transducer to be ${\vec{R}}_{i}^{j}$ and the ${\vec{r}}^{\prime }$ is the position of an arbitrary point, as shown in Figure 1C that is a figure for graphical illustration of the algorithm. The coordinates and relative distances between the point detector and the target position will be more accurate, resulting in even better resolution. *ϑ* is the acoustic velocity in the media. As the difference between the ultrasonic imaging and optical imaging lies solely with the object itself or transducer itself, we only need to double the formula of the *R*_*φ*_(*r*, *θ*) and $\left|{\vec{r}}^{\prime }-{\vec{R}}_{i}^{j}\right|$ , which can be applied to ultrasonic imaging, as shown below. Among them, A is the weight coefficient of the jth unit in the ith detector, which is set to be 1 in this work. Then we can get its pixel value $I\left({\vec{r}}^{\prime }\right)$ . Although this formula is often applied to focused ultrasonic transducers, it can also be applied to plane-based transducers as a special case.

$$I\left(r,\theta \right)=\overset{m}{\underset{\varphi =1}{\sum }}\left(S_{\varphi }\left(2\times R_{\varphi }\left(r,\theta \right)/\vartheta \right)\right)$$ (3)

$$I\left({\vec{r}}^{\prime }\right)=A\times S_{i}\left(2\times |{\vec{r}}^{\prime }-{\vec{R}}_{i}^{j}|/\vartheta \right)$$ (4)

Therefore, if an ultrasonic transducer with an infinite focal length is used in the *X*–*Y* direction, the simple plane form can be adopted, and the algorithm proposed by us can achieve automatic focusing. In the *Z* direction, this can be achieved by focusing the transducer itself. It is also indicated that a line-focused transducer in the *Z* direction could be used to perform high-resolution dynamically focused PA/USE imaging. It also means that targets outside the focal area can be imaged clearly, similarly to synthetic aperture focusing in ultrasonic imaging, or improved endoscopic back-projection, or other dynamic focusing algorithms based on the model, and the SNR can be greatly improved.

### 2.3 Numerical Simulations

In order to comprehensively investigate the effect of the new method proposed in this paper, the following parameters were set as fixed values in all of the numerical simulations: the sampling frequency was 400 MHz, the signal sampling length was 2048, and the ultrasonic center frequency was 40 MHz, the scanning step of the system was 0.5°, and 360°annular scanning was carried out. In the side-view scanning model, each signal in the image is the signal of each point target collected by each transducer unit, which means that the signal can only be detected when the target is facing the mirrored transducer. The original data of point target imaging were simulated in a two-dimensional plane. Seven-point targets were uniformly distributed in the image, with the coordinates of (7,0), (9,0), (11,0), (13,0), (15,0), (17,0), and (19,0), respectively, and the interval of each two points was 2 mm. In order to investigate the image resolution and ultrasonic signal uniformity of the PA/USE experiment with different focal lengths of transducers, four different focal length of transducers were set in simulation: 10, 14, 22 (represent point-focused transducers) and 1,000 mm (similar to the planar detecting surface of line-focused transducer in *X*-*Y* direction), and their diameter are all 4 mm. The proposed PA/USE imaging algorithm and the traditional imaging algorithm were used to calculate and process each set of data, and the reconstruction results of the two imaging methods were analyzed respectively. All those simulations have been done in MATLAB.

### 2.4 Phantom Experiments

Next, a PA/USE experiment was carried out. We designed a hollow cylinder phantom with an outer diameter of 40 mm and an inner diameter of 12 mm to simulate endoscopic imaging in the body cavity. The phantom was made of AGAR powder, water, and fat emulsion in a certain proportion (98 ml water, 2 g AGAR power, 10 ml 10*%* fat emulsion), and the scattering and absorption coefficients were 1 and 0.07 mm^−1^, respectively, relative to the background. Eighteen 0.2 mm-diameter wires were inserted vertically into Phantom 1 as absorbers, and a segment of fresh pig intestinal tissue was inserted into Phantom 2.

**FIGURE 2:**
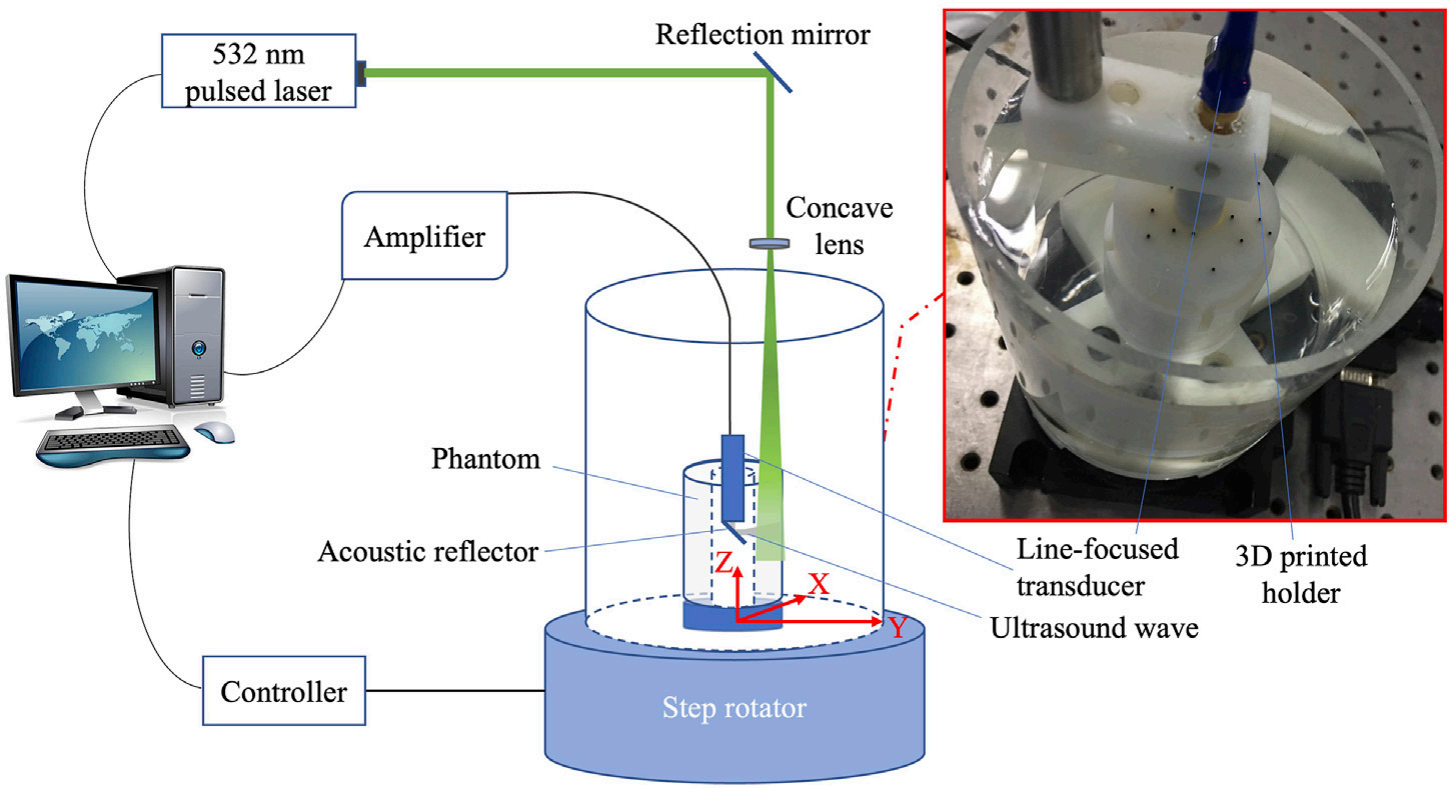
Experimental system diagram of photoacoustic/ultrasonic endoscopy.

The setup of the experimental system of PA/USE with an acoustic resolution is shown in Figure 2. The part shown in the red frame in the figure is the physical image of endoscopic mimetic scanning model. Key parameters of the system are as follows: Q-switched Nd:YAG laser (Nimma-600, Beamtech Co., China) sends out pulsed laser beam at a frequency of 10 Hz and a wavelength of 532 nm. After being reflected by the mirror, the laser beam is diffused to the imitation target by a concave lens to ensure the laser coverage to all targets in the place. The hollow cylinder is placed in a 3D-printed plastic mold and fixed together at the rotating center at the bottom of the water tank. The water tank carrying the cylinder is then fixed at the center of the rotating motor (RAP200, Zolix, China). The phantom, the water tank, and the rotating motor are relatively stationary, while the 3D printed bracket module supports the ultrasonic transducer and is fixed to the center of the hollow model. During the scanning process, the ultrasonic transducer is stationary, while the simulated body rotates in a 360°circle with the water tank and the rotating motor at a certain step length. A stepper motor console (SC300, Zolix, China) is connected to the computer end, and the computer controls the motor rotation through software LabVIEW. The collected signal is transmitted through a radio frequency amplifier (5072 PR, Olympus, 20 dB, 5073 PR, Olympus, 20 dB, Japan) and an acquisition card (LDI400SE, 50 MHz, Diyang, China) stored on the computer. A line-focused ultrasonic transducer with a center frequency of 10 MHz, a bandwidth of 82*%* and a size of 9 mm customized by the merchant was adopted. In the endoscopic scanning experiment, the targets absorb light, and the ultrasonic signal generated by the photoacoustic effect is reflected through the reflector inclined angle to the line-focused transducer and is received. The rotating motor drives the imitation to rotate and scan 720 positions with a step length of 0.5°. In ultrasonic imaging, the laser is turned off and the RF amplifier (5072 PR, Olympus) is used as the ultrasonic transmitter. The feedback ultrasonic signal is received by the transducer and then stored on the computer through the RF amplifier (5072 PR, Olympus, 20dB, 5073 PR, Olympus, 20dB) and the same acquisition card. The acquired photoacoustic signals were averaged four times to eliminate the energy instability and enhance SNR. Finally, the original data (XXX. DAT) files are processed by MATLAB to form images, which are reconstructed with a pixel size of 0.1 mm. The obtained image is a 400 × 400-pixel matrix, and the field of view of reconstructed images is 40 mm × 40 mm in the *X*-*Y* direction.

## 3 Results

### 3.1 Simulation Results

**FIGURE 3:**
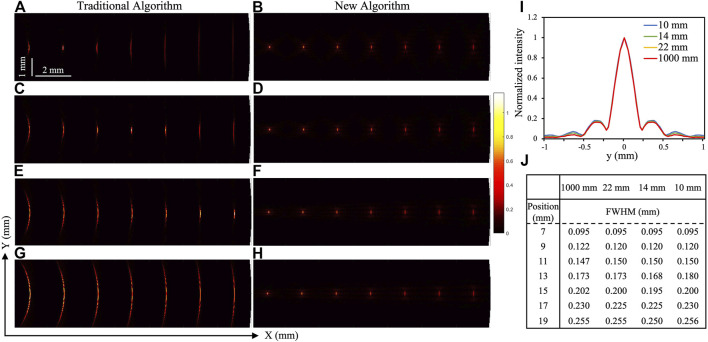
Simulated photoacoustic imaging results in the *X*–*Y* direction. **(A,C,E,G)** The imaging results simulated by traditional PAE reconstruction algorithm under focuses of 10, 14, 22, and 1,000 mm, respectively. **(B,D,F,H)** The imaging results simulated by improved PAE reconstruction algorithm under focuses of 10, 14, 22, and 1,000 mm, respectively. **(I)** The lateral profiles for the seventh target (from left to right; at 19 mm) obtained from (B,D,F,H). The blue, green, yellow, and red lines represent the results of 10, 14, 22, and 1,000 mm, respectively. The amplitudes of these lines are normalized to 1 for comparison. **(J)** The FWHM value of each targets along the *Y* direction in (B,D,F,H).

The simulation results of photoacoustic imaging are shown in Figure 3. Figures 3A,C,E,G show the simulated results by the traditional PAE reconstruction algorithm, while Figures 3B,D,F,H show the simulated results by the proposed algorithm. As seen, when the focal length of the transducer is different, the new algorithm can obtain higher resolutions along the *Y* direction, especially the lateral resolution of the point target outside the focus region. Then, Gaussian fitting curves of the lateral contour of the farthest seventh point target (19,0) under four different focal length transducers are extracted, as shown in Figure 3I. The solid red line represents a focal length of 1,000 mm, and the four curves in the figure tend to coincide, indicating that their image resolutions are very similar. In order to more intuitively compare the resolution comparison of each target under different focal lengths of the transducer in PAE simulation, we calculate the full width at half maximum (FWHM) values of the targets at each position under different focal lengths, as shown in Figure 3J. As seen, the FWHM obtained by the transducer with focal length of 1,000 mm increases almost linearly with the distance from the transducer, which is indeed very similar to the profiles of the other three focal lengths. Therefore, it can be concluded as follows: when the plane ultrasonic transducer is used to replace the focused transducer in the *X*–*Y* direction, in combination with the new algorithm proposed in this paper, higher and more uniform lateral resolution can be obtained especially for those outside the focal region.

**FIGURE 4:**
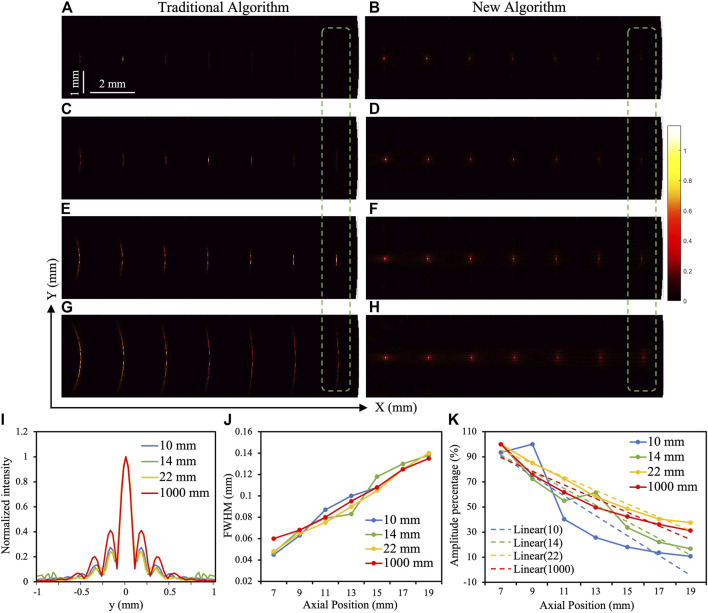
Simulated ultrasonic imaging results in the *X*–*Y* direction. **(A,C,E,G)** The imaging results simulated by traditional USE reconstruction algorithm under focuses of 10, 14, 22, and 1,000 mm, respectively. **(B,D,F,H)** The imaging results simulated by the improved USE reconstruction algorithm under focuses of 10, 14, 22, and 1,000 mm, respectively. **(I)** The lateral profiles for the seventh target (from left to right; at 19 mm) obtained from (B,D,F,H). The blue, green, yellow, and red lines represent the results of 10, 14, 22, and 1,000 mm, respectively. The amplitudes of these lines are normalized to 1 for comparison. **(J)** The FWHM value of each targets along the *Y* direction in (B,D,F,H). **(K)** The contrast curves of seven points under focus of 10, 14, 22, and 1,000 mm, respectively and the linear fitting curves.

Similarly, the simulated ultrasonic imaging results are shown in Figure 4. Figures 4A,C,E,G show the imaging results simulated by the traditional USE reconstruction algorithm, while Figures 4B,D,F,H show the results simulated by the new algorithm. As can be seen, when the focal length of the transducer is different, the new ultrasonic imaging algorithm can be used to obtain higher resolutions than those can be afforded with the traditional algorithm. Figure 4I shows the focal profiles at the seventh point target (19, 0) under four different focal distance transducers, which are applied with the Gaussian curve fitting. The fitted curves are overall quite similar, and even overlap at the main lobe. Figure 4J shows how the extracted FWHM values from (I) chang as a function of target position. Again, it can be seen the trend of linearity is quite similar under different focal lengths.

Although the target at each position can focus on different focal lengths in ultrasonic imaging, it can still be seen that the contrast of the target at each point under different focal lengths is not the same, which is caused by the in-homogeneity of the transmitting sound field of the focused ultrasonic transducer. Especially in the green dotted boxes, the targets in Figures 4A,B are almost not be seen, and as the focus increases, the targets become much clearer and the contrasts also increase obviously. Therefore, we extracted the maximum value of the lateral profile of the target at each point under different focal lengths, set it as y_*i*_, and worked out the amplitude change of the maximum value of the target at each point under each focal length, which can be obtained by the following formula:

$$a=y_{i}/max\left(y_{i}\right)\times 100\%$$ (5)

where *i* = 1–7, respectively representing seven targets with positions ranging from 7 to 19 mm along *Y* direction.

Then, data analysis on the amplitude changes of targets at various points under different focal lengths was conducted and shown in Figure 4K. The solid line is the amplitude change curve of the target at each point under four different focal lengths, and the dashed line is the linear trend line of the corresponding color curve. We got the slopes of the four linear trend lines, as shown in Table 1. When the focal length of the transducer is 10 mm, its slope is the smallest—that is, its trend line is the steepest, which represents the most uneven sound field. Therefore, the target images far from the focus in Figures 4A–D are the blurriest and almost invisible. Therefore, it can be concluded: the larger is the focal length of the transducer, the more uniform is the sound field, and the larger are the target signal contrast and SNR in the ultrasonic imaging, with which the target can be more clearly displayed.

**TABLE 1:** The slope of linear contrast curves at different focal lengths.

Focus (mm)	1,000	22	14	10
Slope	−0.109	−0.107	−0.133	−0.158

To sum up, in both photoacoustic and ultrasonic imaging, a larger focal length of the transducer can not only provide a higher resolution auto-focusing imaging effect, but also obtain a larger radial front and rear target contrast and SNR image. Therefore, the proposed method of using a line-focused transducer with the plane in the *X*–*Y* direction, with integration of the improved back-projection imaging algorithm, has potentials to solve the trade-off between resolution and depth of field in photoacoustic and ultrasonic endoscopic imaging.

### 3.2 Phantom Experiment Results

**FIGURE 5:**
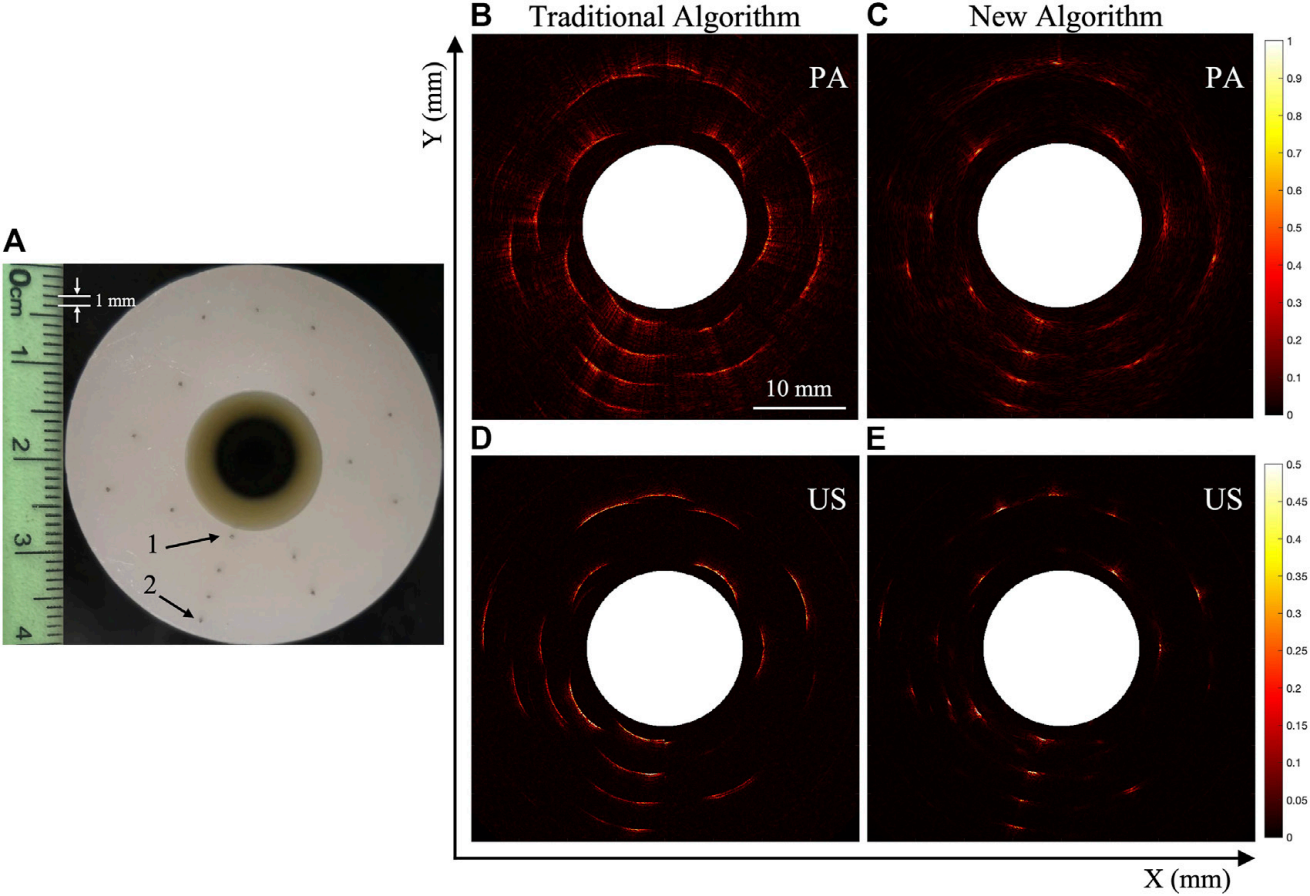
Experimental imaging results of photoacoustic/ultrasonic endoscopy of point targets. **(A)** The photograph of Phantom 1. **(B,C)** The photoacoustic results reconstructed by the traditional algorithm and new algorithm, respectively. **(D,E)** The ultrasonic results reconstructed by the traditional algorithm and new algorithm, respectively.

Figure 5A shows the photograph of Phantom 1 that contains multiple point targets. The photoacoustic and ultrasonic endoscopic scanning imaging experiments were carried out with a 10 MHz line-focused transducer, with both traditional and the new algorithms being used to reconstruct the images. The PA and US results are shown in Figures 5B–E. As seen, the reconstructed PA/USE image quality based on the new algorithm proposed in this paper is overall higher than that based on the traditional imaging algorithm. Two representative point targets were selected, i.e., 1 and 2, in Figures 5C,E, from which the corresponding FWHM values are extracted and shown in Table 2. It can be seen that the resolution of all the point targets reconstructed by the new algorithm based on the line-focused transducer has been considerably improved compared with that obtained with the traditional algorithm for both photoacoustic and ultrasonic images, and the FWHM values of selected 1 and 2 targets are all increased over 4-fold. What’s more, the image quality in the far focal region is also enhanced. These are consistent with what has been observed in simulation.

**TABLE 2:** The FWHM values of target 1 and 2 in photoacoustic and ultrasonic results (Figures 5B–E).

FWHM (mm)						
	PA			US		
Target	Traditional (B)	New (C)	Increased	Traditional (D)	New (E)	Increased
1	5.620	0.956	488%	4.332	0.402	557%
2	7.978	1.290	518%	4.953	0.659	652%

**FIGURE 6:**
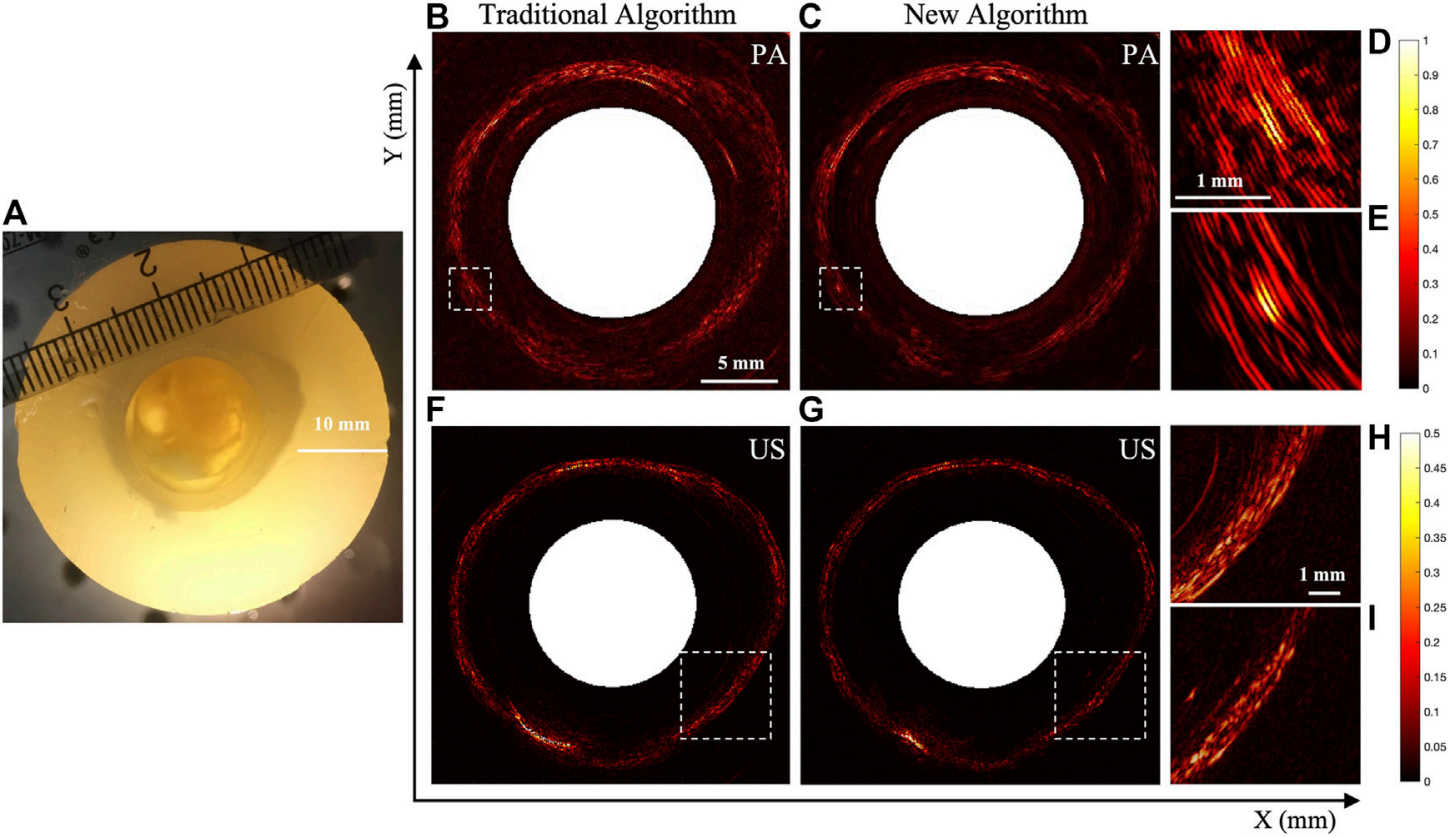
Image reconstruction results of photoacoustic/ultrasonic endoscopy of porcine small intestine. **(A)** Photograph of Phantom 2. **(B-E)** Photoacoustic imaging results **(F-I)** Ultrasonic imaging results. **(B,F)** are obtained with the traditional algorithm, and **(C,G)** are obtained with the new algorithm. **(D,E)** and **(H,I)** are the magnified images of the white boxes in **(B,C)** and **(F,G)**, respectively.

In order to further verify the effect of the proposed method on complex targets, another experimental phantom (Phantom 2) with pig small intestine embedded inside was prepared, and PA/USE experiments were carried out. The results are shown in Figure 6. Among them, Figure 6A shows the photograph of Phantom 2, Figures 6B–E show the photoacoustic endoscopic imaging results, Figures 6F–I show the ultrasonic endoscopic imaging results (field of view is 26 × 26 mm). Note that Figures 6B,F are based on the traditional reconstruction algorithm, and Figures 6C,G are based on the new reconstruction algorithm. Figures 6D,E,H,I are the enlarged version of the white dashed boxes in Figures 6B,C,F,G, respectively, to visualize structure details more clearly. Note that the scale bars are different in the enlarged PA and ultrasonic images. As seen, the details of pig intestinal tissue with the new reconstruction algorithm are more clearly resolved and the imaging SNR is higher in comparison with those that can be obtained with the traditional algorithm.

## 4 Discussion and Conclusion

Dual mode photoacoustic/ultrasonic endoscopy sees wide potential applications in biomedicine. Currently, single focused transducer side scanning method is often adopted in the acoustic-resolution PA/USE imaging. The focal length of the fixed focal transducer is usually limited, and the imaging reconstruction algorithm assumes linear propagation of ultrasonic waves. In this situation, the ultrasonic signals obtained from different angles are directly arranged in the sector of B-scan projection, and hence higher tangential resolution can be obtained only in the vicinity of the focal area. When the targets are away from the focal region, the tangential resolution reduces quickly, which limits the depth of field. Furthermore, in ultrasonic imaging, the point-focused transducer’s transmitting sound field is very uneven, which means that the ultrasonic trigger signal cannot effectively reach the target in the far-focus region, and the feedback ultrasonic signal is correspondingly small or non-existent, severely limiting the depth of field and image contrast. Although some algorithms may be used to improve the resolution, the gap cannot be well recovered for regions away from the focal spot due to the insufficient detection of signals, which poses an inherent contradiction between lateral resolution and depth of field in the current acoustic-resolution PA/USE. To overcome the challenge, a new method based on the use of line-focused transducer is first proposed in this work.

To verify the feasibility of the proposed method, detailed numerical simulation were performed. Four transducers of different numerical aperture (NA) were designed in the simulation and the findings reveal that enhanced BP method can assist all NA transducers in obtaining high-resolution images, implying that this algorithm can disregard NA and achieve automated panoramic depth focusing. Also, results shows that using a line-focused transducer that is approximately flat in the *X*-*Y* direction can not only yield as higher lateral resolution as point-focused one, but also allow clear visualization of targets far away from the focal region, which equivalently increases the depth of field. In this paper, an imaging system was established to mimic PA/USE imaging of point and complex targets in biological tissues. In two-dimensional point target imaging, both photoacoustic and ultrasonic images can provide sub-millimeter lateral resolution within depth of 2 cm, and the farthest off-axis point target can still be clearly displayed. This forms apparent improvement of imaging depth from traditional PA and US endoscopic images. In in vitro porcine small intestine imaging experiments, the details of porcine small intestine are more clearly resolved, and the imaging SNR is considerably higher with the new algorithm. These experimental results demonstrate again that the PA/USE images reconstructed using the new algorithm proposed in this paper can effectively improve the uniformity in the *X*–*Y* direction, and truly realize dynamic focusing in the longitudinal depth direction.

The proposed method and implementation can be easily applied to current acoustic-resolution PA/USE to improve the imaging quality and provide guidance to the design and optimization of other PA/US imaging systems. However, some flaws in this paper exist, such as the use of spatial light alignment lighting rather than optical fiber technology, owing to the fact that the purpose of this paper is to verify the line-focused transducer and the new algorithm to improve the image effect, rather than to design the system or probe. Furthermore, this work concentrates on the development of 2D imaging, with no mention of 3D applications or research. If further engineered, the well-balanced lateral resolution and field depth may also provide innovative inspiration for catheter design and promote the development of PA/USE imaging in clinical applications.

## Data Availability

The raw data supporting the conclusion of this article will be made available by the authors, without undue reservation.
